# Supplementary material for: Altered microRNA Transcriptome in Cultured Human Liver Cells upon Infection with Ebola Virus
Source: Int J Mol Sci. 2021 Apr 6;22(7):3792. doi: 10.3390/ijms22073792 (PMC8038836; doi:10.3390/ijms22073792)
Supplement: Supplementary file 1 [file ijms-22-03792-s001.zip › Supplementary_File/C_ GO_Analysis_Results/16-30nt_go_Makona-96h-Huh7_vs_Control-96h-Huh7_up.mature_mirna_targets/MF_result(Human).html]

| GO.ID | Term | Ontology | Count | Pop.Hits | List.Total | Pop.Total | Fold.Enrichment | Pvalue | FDR | Enrichment.Score | Gene.Ratio | GENES |
| --- | --- | --- | --- | --- | --- | --- | --- | --- | --- | --- | --- | --- |
| GO:0042605 | peptide antigen binding | Molecular function | 4 | 28 | 147 | 17548 | 17.0534499514091 | 8.27664394077953e-05 | 0.107265305472503 | 4.08214572764286 | 0.0272108843537415 | CLEC4M//DHCR24//CD209//HLA-DPB1// |
| GO:0003700 | DNA binding transcription factor activity | Molecular function | 24 | 1551 | 147 | 17548 | 1.84718219976579 | 0.00239550785126412 | 0.791072678256733 | 2.62060240140567 | 0.163265306122449 | WDHD1//FOXP4//CUX2//EBF3//ALX3//NR6A1//HOXA9//MITF//PAX7//QRICH1//RARB//ALX4//HIF3A//NCOA1//CREB5//FUBP3//PURB//KAT7//ZNF320//AFF3//RNF4//ZNF329//LZTR1//ZNF251// |
| GO:0015279 | store-operated calcium channel activity | Molecular function | 2 | 10 | 147 | 17548 | 23.8748299319728 | 0.00300124751211665 | 0.791072678256733 | 2.52269818694307 | 0.0136054421768707 | TRPC3//TRPC5// |
| GO:0051427 | hormone receptor binding | Molecular function | 6 | 179 | 147 | 17548 | 4.00136814502337 | 0.00400367643394577 | 0.791072678256733 | 2.39754102825318 | 0.0408163265306122 | NCOA1//TAF11//RARB//RNF4//NCOA4//NPPC// |
| GO:0052629 | phosphatidylinositol-3,5-bisphosphate 3-phosphatase activity | Molecular function | 2 | 12 | 147 | 17548 | 19.8956916099773 | 0.00435372887308991 | 0.791072678256733 | 2.36113861998653 | 0.0136054421768707 | MTM1//SYNJ1// |
| GO:0140110 | transcription regulator activity | Molecular function | 27 | 1921 | 147 | 17548 | 1.67782511234582 | 0.00484509470028107 | 0.791072678256733 | 2.3146977299842 | 0.183673469387755 | WDHD1//FOXP4//CUX2//EBF3//ALX3//NR6A1//HOXA9//MITF//PAX7//QRICH1//RARB//ALX4//HIF3A//NCOA1//CREB5//FUBP3//PURB//KAT7//ZNF320//AFF3//RNF4//ZNF329//LZTR1//ZNF251//RIPK3//TAF11//NCOA4// |
| GO:0070679 | inositol 1,4,5 trisphosphate binding | Molecular function | 2 | 13 | 147 | 17548 | 18.3652537938252 | 0.00511715406734687 | 0.791072678256733 | 2.29097150708665 | 0.0136054421768707 | TRPC3//TRPC5// |
| GO:0050681 | androgen receptor binding | Molecular function | 3 | 43 | 147 | 17548 | 8.32842904603702 | 0.00556293614546485 | 0.791072678256733 | 2.25469592510886 | 0.0204081632653061 | RNF4//NCOA4//NCOA1// |
| GO:0004438 | phosphatidylinositol-3-phosphatase activity | Molecular function | 2 | 15 | 147 | 17548 | 15.9165532879819 | 0.00681335217513988 | 0.791072678256733 | 2.16663916224701 | 0.0136054421768707 | MTM1//SYNJ1// |
| GO:0052744 | phosphatidylinositol monophosphate phosphatase activity | Molecular function | 2 | 15 | 147 | 17548 | 15.9165532879819 | 0.00681335217513988 | 0.791072678256733 | 2.16663916224701 | 0.0136054421768707 | MTM1//SYNJ1// |
| GO:0106018 | phosphatidylinositol-3,5-bisphosphate phosphatase activity | Molecular function | 2 | 15 | 147 | 17548 | 15.9165532879819 | 0.00681335217513988 | 0.791072678256733 | 2.16663916224701 | 0.0136054421768707 | SYNJ1//MTM1// |
| GO:0016307 | phosphatidylinositol phosphate kinase activity | Molecular function | 2 | 17 | 147 | 17548 | 14.0440176070428 | 0.00872879818121055 | 0.791072678256733 | 2.05904554772611 | 0.0136054421768707 | PIP5K1A//PIP5K1C// |
| GO:0042834 | peptidoglycan binding | Molecular function | 2 | 17 | 147 | 17548 | 14.0440176070428 | 0.00872879818121055 | 0.791072678256733 | 2.05904554772611 | 0.0136054421768707 | NOD1//PGLYRP4// |
| GO:0035257 | nuclear hormone receptor binding | Molecular function | 5 | 151 | 147 | 17548 | 3.95278641257828 | 0.00887905772363506 | 0.791072678256733 | 2.05163312061094 | 0.0340136054421769 | NCOA1//TAF11//RARB//RNF4//NCOA4// |
| GO:0003677 | DNA binding | Molecular function | 32 | 2520 | 147 | 17548 | 1.51586221790303 | 0.00959724119208133 | 0.791072678256733 | 2.01785359063191 | 0.217687074829932 | RARB//CUX2//H2AFY2//PURB//ALX4//EBF3//NR6A1//HOXA9//MITF//FUBP3//KAT7//AFF3//SLTM//FOXP4//ALX3//PAX7//ZNF329//CDK12//WDHD1//ZNF488//ZNF597//ZNF320//KDM2A//YBX2//QRICH1//RNF4//HIF3A//ZC3H12A//NCOA1//ZNF251//CREB5//TRAF4// |
| GO:0005537 | mannose binding | Molecular function | 2 | 18 | 147 | 17548 | 13.2637944066515 | 0.00976632936119424 | 0.791072678256733 | 2.01026863359099 | 0.0136054421768707 | CLEC4M//CD209// |
| GO:0061783 | peptidoglycan muralytic activity | Molecular function | 2 | 19 | 147 | 17548 | 12.5656999641962 | 0.0108558049900399 | 0.822412648749125 | 1.96433796679547 | 0.0136054421768707 | LYZL6//PGLYRP4// |
| GO:0000981 | RNA polymerase II transcription factor activity, sequence-specific DNA binding | Molecular function | 17 | 1108 | 147 | 17548 | 1.83155283774159 | 0.0114223978992934 | 0.822412648749125 | 1.94224271525708 | 0.115646258503401 | PAX7//EBF3//NR6A1//MITF//FUBP3//CUX2//PURB//ALX4//RARB//WDHD1//FOXP4//ALX3//HOXA9//QRICH1//HIF3A//NCOA1//CREB5// |
| GO:0043169 | cation binding | Molecular function | 47 | 4225 | 147 | 17548 | 1.32794912047659 | 0.0178189243691446 | 1 | 1.74911851548681 | 0.319727891156463 | MAP3K8//YDJC//PCK1//EDIL3//DGKA//DLL4//NECAB3//CDH24//THBS1//TCHH//PRRG3//KAT7//USP51//KDM2A//NR6A1//PGLYRP4//RARB//RNF4//TIMP2//ZNF185//TRAF4//SATL1//CLEC4M//FOXP4//ZNF488//MOB3A//ZNF597//MOB3C//ZNF320//DZIP1//EBF3//SUMF2//CD209//RFESD//DBR1//PDE1B//PRKAA2//BAK1//MUL1//ZNF329//ZC3H12A//PRR3//ZNF251//IDI2//CACNA2D4//CREB5//EXOG// |
| GO:0046872 | metal ion binding | Molecular function | 46 | 4131 | 147 | 17548 | 1.32926915622216 | 0.0189341911468008 | 1 | 1.72275324285286 | 0.312925170068027 | MAP3K8//YDJC//PCK1//EDIL3//DGKA//DLL4//NECAB3//CDH24//THBS1//TCHH//PRRG3//KAT7//USP51//KDM2A//NR6A1//PGLYRP4//RARB//RNF4//TIMP2//ZNF185//TRAF4//CLEC4M//FOXP4//ZNF488//MOB3A//ZNF597//MOB3C//ZNF320//DZIP1//EBF3//SUMF2//CD209//RFESD//DBR1//PDE1B//PRKAA2//BAK1//MUL1//ZNF329//ZC3H12A//PRR3//ZNF251//IDI2//CACNA2D4//CREB5//EXOG// |
| GO:0072509 | divalent inorganic cation transmembrane transporter activity | Molecular function | 2 | 26 | 147 | 17548 | 9.18262689691261 | 0.0198598537375319 | 1 | 1.70202395429062 | 0.0136054421768707 | SLC30A2//SLC41A1// |
| GO:0034593 | phosphatidylinositol bisphosphate phosphatase activity | Molecular function | 2 | 27 | 147 | 17548 | 8.84252960443437 | 0.0213323464457033 | 1 | 1.67096137181855 | 0.0136054421768707 | SYNJ1//MTM1// |
| GO:0046983 | protein dimerization activity | Molecular function | 18 | 1286 | 147 | 17548 | 1.67086679150665 | 0.0219080961566777 | 1 | 1.65939536158008 | 0.122448979591837 | NOD1//FKBP1A//NR6A1//NPPC//ACPP//BAK1//CDH24//TFRC//GCLM//H2AFY2//PGLYRP4//ALX4//STX1A//TAF11//EBF3//MITF//HIF3A//NCOA1// |
| GO:0001618 | virus receptor activity | Molecular function | 3 | 74 | 147 | 17548 | 4.83949255377827 | 0.0242286243438407 | 1 | 1.61567124359421 | 0.0204081632653061 | CLEC4M//CD209//TFRC// |
| GO:0104005 | hijacked molecular function | Molecular function | 3 | 74 | 147 | 17548 | 4.83949255377827 | 0.0242286243438407 | 1 | 1.61567124359421 | 0.0204081632653061 | CLEC4M//CD209//TFRC// |
| GO:0005251 | delayed rectifier potassium channel activity | Molecular function | 2 | 29 | 147 | 17548 | 8.23269997654234 | 0.0244084411712196 | 1 | 1.61245995562398 | 0.0136054421768707 | KCNC4//KCNS1// |
| GO:0016411 | acylglycerol O-acyltransferase activity | Molecular function | 2 | 29 | 147 | 17548 | 8.23269997654234 | 0.0244084411712196 | 1 | 1.61245995562398 | 0.0136054421768707 | AGPAT4//MOGAT3// |
| GO:0052866 | phosphatidylinositol phosphate phosphatase activity | Molecular function | 2 | 30 | 147 | 17548 | 7.95827664399093 | 0.0260103947869704 | 1 | 1.58485305601245 | 0.0136054421768707 | MTM1//SYNJ1// |
| GO:0005164 | tumor necrosis factor receptor binding | Molecular function | 2 | 31 | 147 | 17548 | 7.70155804257187 | 0.0276538712604168 | 1 | 1.55824406329775 | 0.0136054421768707 | LTA//TRAF4// |
| GO:0003690 | double-stranded DNA binding | Molecular function | 13 | 860 | 147 | 17548 | 1.80449295997469 | 0.0286397880763643 | 1 | 1.54303019996775 | 0.0884353741496599 | RARB//CUX2//H2AFY2//PURB//ALX4//EBF3//NR6A1//HOXA9//MITF//FUBP3//KAT7//AFF3//SLTM// |
| GO:0043565 | sequence-specific DNA binding | Molecular function | 16 | 1139 | 147 | 17548 | 1.67689762472153 | 0.0291901658331554 | 1 | 1.53476343772114 | 0.108843537414966 | RARB//CUX2//H2AFY2//PURB//ALX4//EBF3//NR6A1//HOXA9//MITF//FUBP3//KAT7//FOXP4//ALX3//PAX7//ZNF329//SLTM// |
| GO:0022890 | inorganic cation transmembrane transporter activity | Molecular function | 10 | 601 | 147 | 17548 | 1.98625872978143 | 0.0299675717741943 | 1 | 1.5233484458362 | 0.0680272108843537 | COX4I1//CACNA2D4//KCNC4//KCNS1//TRPC3//TRPC5//SLC30A2//SLC41A1//TFRC//ATP6V0E1// |
| GO:0008080 | N-acetyltransferase activity | Molecular function | 3 | 86 | 147 | 17548 | 4.16421452301851 | 0.0355982027185642 | 1 | 1.44857192812583 | 0.0204081632653061 | SATL1//KAT7//NCOA1// |
| GO:0016747 | transferase activity, transferring acyl groups other than amino-acyl groups | Molecular function | 5 | 221 | 147 | 17548 | 2.700772616739 | 0.0385030205134742 | 1 | 1.41450519929984 | 0.0340136054421769 | AGPAT4//MOGAT3//SATL1//KAT7//NCOA1// |
| GO:0046873 | metal ion transmembrane transporter activity | Molecular function | 8 | 457 | 147 | 17548 | 2.0897006505009 | 0.0387251866468737 | 1 | 1.41200648035993 | 0.054421768707483 | CACNA2D4//KCNC4//KCNS1//TRPC3//TRPC5//SLC30A2//SLC41A1//TFRC// |
| GO:0035258 | steroid hormone receptor binding | Molecular function | 3 | 89 | 147 | 17548 | 4.02384774134373 | 0.0388012911029217 | 1 | 1.41115382312885 | 0.0204081632653061 | NCOA1//RNF4//NCOA4// |
| GO:0043167 | ion binding | Molecular function | 62 | 6159 | 147 | 17548 | 1.20168814400253 | 0.0441011034390022 | 1 | 1.35555054405882 | 0.421768707482993 | ACBD7//MAP3K8//YDJC//PCK1//THBS1//EDIL3//DGKA//DLL4//NECAB3//CDH24//TCHH//PRRG3//NOD1//RIPK3//KIF1B//PIP5K1C//CDK12//ETNK2//PRKAA2//CAMK1G//MCCC2//PIP5K1A//KAT7//USP51//KDM2A//NR6A1//PGLYRP4//RARB//RNF4//TIMP2//ZNF185//TRAF4//SATL1//RUNDC3B//MTM1//CLEC4M//FOXP4//ZNF488//MOB3A//ZNF597//MOB3C//ZNF320//DZIP1//EBF3//SUMF2//CD209//RFESD//DBR1//PDE1B//BAK1//MUL1//ZNF329//ZC3H12A//PRR3//ZNF251//IDI2//CACNA2D4//CREB5//EXOG//DHCR24//TRPC3//TRPC5// |
| GO:0046915 | transition metal ion transmembrane transporter activity | Molecular function | 2 | 40 | 147 | 17548 | 5.9687074829932 | 0.0441853724462058 | 1 | 1.35472147991119 | 0.0136054421768707 | SLC30A2//TFRC// |
| GO:0005515 | protein binding | Molecular function | 107 | 11565 | 147 | 17548 | 1.10445603230484 | 0.044540017184212 | 1 | 1.35124961974457 | 0.727891156462585 | STX1A//THBS1//TIMP2//PPP1R13B//TNS1//TRPC5//LTA//NPPC//DLL4//SMO//FKBP1A//TRAF4//EDIL3//FAS//PDE1B//CAMK1G//KIF1B//CDH24//GPX3//CDK12//PURB//RARB//RNF4//TAF11//NCOA1//DHCR24//HOXA9//PIP5K1A//SHISA6//NMD3//BAK1//MUL1//CUL2//RGS7//CLTCL1//GCLM//USP51//NOD1//RIPK3//GJB2//ACPP//TFRC//KCTD15//NR6A1//H2AFY2//PGLYRP4//ALX4//EBF3//MITF//HIF3A//CLEC4M//NCOA4//ZNF185//WDR1//WDHD1//CDK2AP1//KAT7//FOXP4//ZNF488//TMEM170A//TPRA1//MAP3K8//COX4I1//TMEM199//SPC24//MOB3C//ZNF320//F2RL2//DZIP1//KDM2A//MAPKBP1//LARP4B//PIP5K1C//COX18//KRT6C//CD274//CD209//RFESD//KCNS1//MTM1//PMP22//QRICH1//LRRC20//TMEM100//PRKAA2//NRIP3//AGPAT4//PTPN7//NECAB3//MCCC2//TACC1//PRDX2//TRPC3//SLC30A2//LAPTM5//ZNF329//ZC3H12A//PRR3//KRTAP4-12//RASSF4//PYROXD2//SYNJ1//FUBP3//COL23A1//CREB5//RASSF2//MFAP3L// |
| GO:0005488 | binding | Molecular function | 133 | 15006 | 147 | 17548 | 1.05802758261775 | 0.0493632125396251 | 1 | 1.30659658433073 | 0.904761904761905 | ACBD7//STX1A//MAP3K8//YDJC//PCK1//RARB//CUX2//H2AFY2//PURB//ALX4//EBF3//NR6A1//HOXA9//MITF//FUBP3//THBS1//TIMP2//PPP1R13B//DZIP1//EXOG//WDHD1//ZNF488//ZNF597//ZNF320//KDM2A//YBX2//QRICH1//RNF4//HIF3A//ZNF329//ZC3H12A//NCOA1//ZNF251//CREB5//TRAF4//USP51//PRKAA2//KAT7//AFF3//SLTM//MAPKBP1//LARP4B//NOVA2//NMD3//DBR1//TFRC//TNS1//PRR3//SYNJ1//TRPC5//LTA//NPPC//DLL4//SMO//FKBP1A//EDIL3//DGKA//NECAB3//CDH24//TCHH//PRRG3//CLEC4M//NOD1//RIPK3//FOXP4//TMEM170A//TPRA1//COX4I1//TMEM199//SPC24//MOB3C//F2RL2//KIF1B//PIP5K1C//COX18//KRT6C//GPX3//CD274//CD209//RFESD//FAS//KCNS1//MTM1//PDE1B//CDK12//PMP22//ACPP//LRRC20//TMEM100//NRIP3//AGPAT4//PTPN7//BAK1//MCCC2//TACC1//TAF11//PRDX2//TRPC3//SLC30A2//LAPTM5//KCTD15//MUL1//CDK2AP1//CLTCL1//KRTAP4-12//RASSF4//PIP5K1A//CUL2//PYROXD2//COL23A1//RASSF2//MFAP3L//CAMK1G//ETNK2//PGLYRP4//ZNF185//SATL1//DHCR24//SHISA6//RGS7//RUNDC3B//GCLM//HLA-DPB1//GJB2//ALX3//PAX7//MOB3A//SUMF2//IDI2//CACNA2D4//H6PD//NCOA4//WDR1// |
